# Assessment of National Early Warning Score 2 as a Tool to Predict the Outcome of COVID-19 Patients on Admission

**DOI:** 10.7759/cureus.21164

**Published:** 2022-01-12

**Authors:** Balchandra Chikhalkar, Dhruv Gosain, Shruti Gaikwad, Rohit Deshmukh

**Affiliations:** 1 Forensic Medicine Department, Grant Medical College and Sir Jamshedjee Jeejeebhoy (JJ) Group of Hospitals, Mumbai, IND; 2 Emergency Department, Grant Medical College and Sir Jamshedjee Jeejeebhoy (JJ) Group of Hospitals, Mumbai, IND

**Keywords:** covid prognosis, predicting mortality, triage, news 2 score, covid-19

## Abstract

Introduction: The ongoing pandemic due to coronavirus disease 2019 (COVID-19) has put tremendous strain on the healthcare system around the world. There is a paucity of data describing the role of National Early Warning Score 2 (NEWS2) in the assessment of COVID-19 cases. This study aimed at identifying NEWS2 calculated on admission as a valuable tool for risk stratification and prediction of in-hospital mortality in COVID-19 patients.

Materials and method: This prospective, observational study included 814 confirmed COVID-19 cases and was conducted over a period of three months. Vital parameters were assessed and NEWS2 was calculated on admission. Data were entered in excel format and statistical analysis was done in Python 3.8 statistical software (Wilmington, DE: Python Software Foundation). Pearson's chi-squared test was used following which a significant NEWS2 cut-off score to predict in-hospital mortality was determined by means of receiver operating characteristic (ROC) curve.

Results: Mortality of 9.09% was noted and correlations were made with age, comorbidity, and NEWS2 score. For in-hospital deaths, comorbidities were present in 66.21% of patients, the mean age was 60.14 years, and average NEWS2 score was 9. For discharged patients only 21.89% had comorbidities, mean age was 42.96 years, and average NEWS2 score was 1.17. NEWS2 score of ≥ 6 had a sensitivity of 93.24% and specificity of 98.91%, and hence was a statistically significant cut-off value for predicting mortality on admission.

Conclusion:Age, presence of comorbidities, and NEWS2 have a positive correlation with mortality in COVID-19 patients. NEWS2 score being easy, reliable, and quick to calculate, should be used to triage these patients on admission. Scores ≥ 6 should be considered to have a higher risk of adverse outcomes and hence should be managed prudently along with clinical judgment.

## Introduction

Coronavirus disease 2019 (COVID-19) is an infectious disease caused by severe acute respiratory syndrome coronavirus 2 (SARS-CoV-2). The first confirmed case was reported in Wuhan, China in December 2019 [1]. The disease has since spread worldwide, leading to an ongoing pandemic and has added a burden on the healthcare system worldwide. Contact with or inhalation of contaminated droplets is thought to be the mode of transmission, while the incubation period spans from two to 14 days (median: five days) [2]. The virus actively replicates in tissues of the upper respiratory tract and can be isolated from these regions [3]. Fever, cough, sore throat, dyspnea, and lethargy are frequent symptoms [4]. The condition is usually asymptomatic, but it can advance to pneumonia, acute respiratory distress syndrome (ARDS), and multi-organ failure in some individuals [5]. A higher viral load has been found in patients who develop severe symptoms [6].

Comorbidities commonly found in COVID-19 patients are hypertension, diabetes, chronic lung diseases, and cardiovascular diseases; hypertension being the commonest [7,8]. It has been observed that diabetic [9] or hypertensive patients are more likely to develop severe symptoms [10]. Age > 60 years has been noted to contribute independently in developing severe disease [11,12]. Furthermore, a study suggests that the risk of mortality is increased significantly in patients aged 60 years and older, who have associated comorbidities [7]. Therefore, patients who are at risk of developing a progressive disease should be discerned in the emergency department to prevent mortality. While raised inflammatory markers, deranged red cell distribution width (RDW), neutrophil-leucocyte ratio (NLR), and lactate dehydrogenase (LDH) have been identified as prognostic predictors of severe disease in COVID-19 patients; these blood reports are not readily available during an emergency when the patient first visits the hospital [13]. Various scoring systems (e.g., National Early Warning Score 2 {NEWS2}, CURB-65, systemic inflammatory response syndrome) can facilitate the early identification of deteriorating patients [14-17]. However, concrete data on the effectiveness of such scores in guiding treatment decisions in COVID-19 patients is currently lacking [18]. The Royal College of Physicians (RCP) has identified NEWS2 as a method for standardizing the evaluation and management of acute illness [19]. The score is based on six physiological parameters: (i) respiratory rate, (ii) oxygen saturation and air or oxygen supplementation, (iii) systolic blood pressure, (iv) temperature, (v) pulse rate, and (vi) consciousness level on the alert, voice, pain, unresponsive (AVPU) scale [20]. NEWS2 was classified into three clinical risk categories by the RCP: low (0-4), medium (5-6), and high (≥ 7) [20].

There is a dire need for a tool that can not only identify patients with severe disease and high risk of adverse outcomes but also stratify them. In the face of a shortage of personnel and equipment, it will help in prompt management and in using the existing resources more efficiently. As the parameters utilized to calculate the NEWS2 score can be determined by mere examination and by using equipment, which is easily accessible, it can prove to be useful in the emergency department. Hence, we aim to ascertain whether NEWS2 calculated on admission in the emergency department helps us with risk stratification and predicting mortality in COVID-19 patients.

## Materials and methods

This is a prospective, observational research conducted at a tertiary care COVID-19-dedicated hospital, over a span of three months. This study was approved by institutional ethics committee. We aimed to determine whether NEWS2 calculated on admission is a valuable tool for predicting severe outcomes in COVID-19 patients and if an acceptable cut-off value can be obtained to predict in-hospital mortality.

Real-time reverse transcription-polymerase chain reaction (RT-PCR) detected COVID-19-positive patients admitted via the emergency department, during a period of three months, were recruited for this study. These patients were diagnosed as per the Indian Council of Medical Research guidelines and National Institute of Virology instructions with the help of real-time fluorescent RT-PCR kit testing of nasopharyngeal swabs [21,22].

NEWS2 was routinely utilized in the hospital's emergency department and medical wards to stratify patient risk and assign patients to the most suitable level of treatment (ward or intensive care). Patients were triaged on admission, and their age, sex, comorbidities, symptoms, and vital signs were noted. Data were collected over three months. The primary endpoint was identified as intra-hospital death/discharge. Hence, patients transferred out to other hospitals were excluded as their outcomes were unknown. Also, patients with symptoms but no COVID-positive reports were excluded from the study and ultimately, a total of 814 patients were eligible.

**Figure 1:**
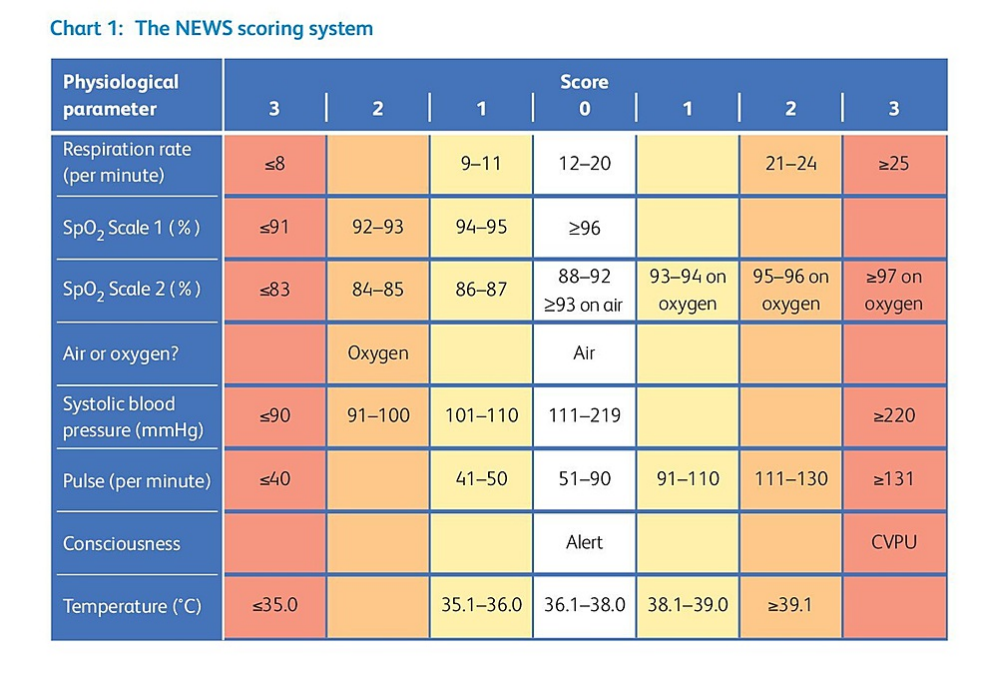
The NEWS scoring system. CVPU: new confusion, voice, pain, unresponsive; SpO2: oxygen saturation Reproduced from Royal College of Physicians. National Early Warning Score (NEWS) 2: Standardising the assessment of acute illness severity in the NHS. Updated report of a working party. London: RCP, 2017 [19]. In order to encourage as many people as possible to use the material in their publication, there is no copyright restriction [19]. High-quality versions of the charts and their explanatory text are available to download, photocopy or print directly from www.rcplondon.ac.uk/national-early-warning-score [19].

**Figure 2:**
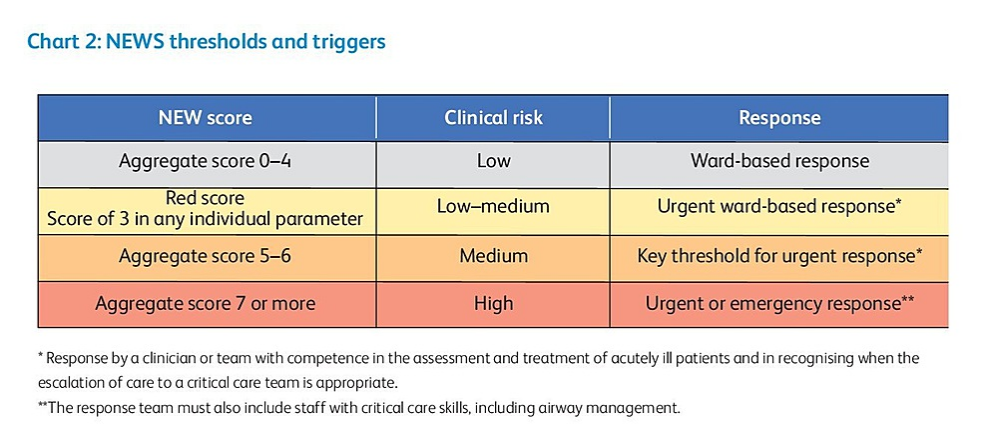
NEWS thresholds and triggers. Reproduced from Royal College of Physicians. National Early Warning Score (NEWS) 2: Standardising the assessment of acute illness severity in the NHS. Updated report of a working party. London: RCP, 2017 [19]. In order to encourage as many people as possible to use the material in their publication, there is no copyright restriction [19]. High-quality versions of the charts and their explanatory text are available to download, photocopy or print directly from www.rcplondon.ac.uk/national-early-warning-score [19].

The NEWS2 score is generated by adding the scores of six physiological parameters: oxygen saturation, respiration rate, pulse rate, systolic blood pressure, level of consciousness or new confusion, and body temperature, each with a 0-3 point scale [23]. The differences in the parameters are reflected in the score. Patients needing further supplementary oxygen are allocated additional two points [23]. The National Institute for Health and Care Excellence (NICE) recommends NEWS2 score in its guidelines for the treatment of COVID-19 patients in intensive care [23]. NEWS2 is divided into three risk groups: low risk (0-4), medium risk (5-6), and high risk (≥ 7), wherein a score of 5 or 6 is regarded a crucial cut-off that may indicate clinical worsening and should necessitate an immediate reaction by a clinician or a team with expertise in evaluating and managing severely unwell individuals (Figures 1, 2) [24].

In-hospital death/discharge was the endpoint till when the patients were followed up. In-hospital mortality was identified as death occurring while hospitalized due to any cause, including COVID-19 infection. Data collected were added to excel format. Graphs and charts were made to explain the demographic distribution of our data set. All statistical analysis was done in Python 3.8 statistical software (Wilmington, DE: Python Software Foundation). Initially, we used Pearson's chi-squared test to determine if outcome was dependent or independent of NEWS2 score (Pearson's chi-squared test is a statistical test applied to sets of categorical data to evaluate how likely it is that any observed difference between the sets arose by chance). To evaluate which feature was a good predictor of the outcome, we used logistic regression in python with the Scikit-learn library. We also used the Python Scikit-learn library to generate specificity, sensitivity, F-1 score, negative predictive value, and positive predictive values with 95% confidence intervals (CIs) to evaluate the ability of NEWS2 score at different cut-offs to predict in-hospital mortality. We evaluated every NEWS2 score cut-off using area under the receiver operating characteristic (AUROC) performance metric. Pandas and NumPy libraries in python were used for data manipulation and analysis. Matplotlib library was used to plot graphs.

## Results

A total of 814 (564 males, 250 females) eligible patients were enrolled in the study over three months. NEWS2 was calculated on admission and patient age, comorbidities, and symptoms were noted. Outcomes with 74 deaths and 740 discharges (9.09% mortality) were observed. While the average NEWS2 score in discharged patients was 1.17, those who succumbed had a significantly higher score of 9 (Table 1). Age and sex distribution of indoor patients can be seen in Table 2.

**Table 1:** Mean age and mean NEWS2 score observed among total patients, discharged patients and in-hospital deaths. NEWS2: National Early Warning Score 2

	Mean age (years)	Mean NEWS2 score
Total patients	44.52	1.88
Discharges	42.96	1.17
Deaths	60.14	9

**Table 2:** Age and sex distribution of indoor COVID-19 patients. COVID-19: coronavirus disease 2019

Age (years)	Female	Male
18-20	15	17
21-30	44	137
31-40	50	126
41-50	38	106
51-60	40	84
61-70	27	53
71-80	23	25
81-90	10	13
91-100	3	3

Most number of discharges were observed in the 21-30-year age group (n=180) and NEWS2 score range of 0-5 (n=715). The highest mortality was observed in the age group of 61-70 years (n=22) and NEWS2 range of 5-10 (n=46) (Tables 3, 4).

**Table 3:** Outcome across age groups of indoor COVID-19 patients. COVID-19: coronavirus disease 2019

Age (years)	Discharges	Deaths
18-20	32	0
21-30	180	1
31-40	168	8
41-50	132	12
51-60	111	13
61-70	58	22
71-80	36	12
81-90	19	4
91-100	4	2

**Table 4:** NEWS2 distribution of indoor COVID-19 patients. NEWS2: National Early Warning Score 2; COVID-19: coronavirus disease 2019

NEWS-2 score	Discharges	Deaths
0-5	715	2
5-10	25	46
10-15	0	22
15-20	0	4

A total of 250 females and 564 males were included in the study. Out of 250 females, 229 were discharged (average NEWS2: 1.35), while 21 succumbed (average NEWS2: 8.47). Of the 564 males included, 511 were discharged (average NEWS2: 1.09) and 53 of them expired (average NEWS2: 9.21). Female mortality was 8.4%, while male mortality was 9.39%.

In patients who got discharged, 78.10% had no associated comorbidities, while for patients who succumbed, the percentage was 33.78%. Rest were patients with single or multiple comorbidities. Diabetes mellitus and hypertension were the most prevalent (36.48% each) in those who succumbed to COVID-19 (Table 5).

**Table 5:** Distribution of comorbidities in indoor COVID-19 patients. COPD: chronic obstructive pulmonary disease; TB: tuberculosis; COVID-19: coronavirus disease 2019

Comorbidities	Discharges	Deaths	Total
None	578	25	603
Diabetes mellitus	75	27	102
Hypertension	88	27	115
Ischemic heart disease	9	2	11
Pulmonary TB	13	1	14
Hypothyroidism	12	3	15
Asthma/COPD	8	4	12
Chronic kidney disease	2	1	3
HIV	8	2	10
Chronic liver disease	2	1	3
Carcinoma	2	2	4

Chi-squared test at 95% for NEWS2 score and outcome (discharge or death) had a p-value < 0.05, and therefore we concluded that mortality is dependent on National Early Warning Score 2 (NEWS2) in the dataset. Using logistic regression, adding more features such as age or gender or comorbidities did not improve the performance (area under the curve {AUC}: 0.97) as compared to performance of NEWS2 alone (AUC: 0.99). Therefore, we proceeded to evaluate NEWS2 at various cut-offs to determine the ideal cut-off score to predict in-hospital mortality (Table 6).

**Table 6:** Comparison between sensitivity, specificity, precision, F-1 score, and accuracy for NEWS2 scores 3 to 9. NEWS2: National Early Warning Score 2

NEWS2 score ≥ x	Sensitivity	Specificity	Precision	F-1 score	Accuracy
3	0.986486486	0.848648649	0.394594595	0.563706564	0.861179361
4	0.986486486	0.925675676	0.5703125	0.722772277	0.931203931
5	0.972972973	0.966216216	0.742268041	0.842105263	0.966830467
6	0.932432432	0.989189189	0.896103896	0.913907285	0.984029484
7	0.864864865	0.997297297	0.96969697	0.914285714	0.985257985
8	0.689189189	0.998648649	0.980769231	0.80952381	0.970515971
9	0.5	1	1	0.666666667	0.954545455

**Figure 3:**
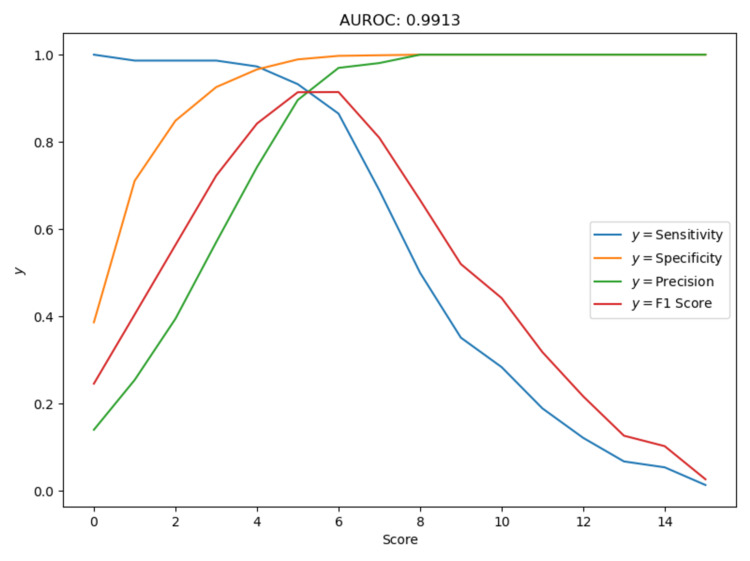
Receiver operator characteristic curves depicting the ability of NEWS2 scores calculated in the emergency department on admission to predict COVID-19 in-hospital mortality. AUROC: area under the receiver operator characteristic curve; NEWS2: National Early Warning Score 2; COVID-19: coronavirus disease 2019

Figure 3 shows ROC curve for prediction of mortality using NEWS2 on admission. Out of each score of the NEWS2, scores ≥5 or ≥ 6 are significant. NEWS2 score ≥ 5 has sensitivity of 0.972, specificity of 0.966, precision of 0.74, F-1 score of 0.84, and accuracy of 0.96. For NEWS2 score ≥ 6 sensitivity is at 0.932, specificity at 0.989, precision at 0.896, F-1 score at 0.91, and accuracy of 0.98.

## Discussion

In the midst of the ongoing pandemic, it's important to identify patients who are at risk of developing severe illness ahead of time so that prompt decisions can be made about the level of in-hospital treatment they should receive. Timely identification is crucial as coronavirus pneumonia is typically lethal due to its fast development. Due to the extremely infectious nature of this viral illness, medical personnel are needed to wear personal protective equipment, resulting in less interaction with COVID-19 patients. Such constraints may cause delays in identifying patients who are at risk. NEWS2 enables intense surveillance by identifying people at risk of developing a life-threatening illness.

The role of NEWS2 in COVID-19 has not yet been thoroughly investigated, even though it is commonly used in clinical practice. According to the advice of Royal College of Physicians, NEWS2 should be utilized while monitoring COVID-19 patients, although, further evaluation should be made if there is any rise in oxygen requirements [20]. Using NEWS2 will make sure that patients who are worsening or at risk of deterioration are recognized and examined as soon as possible by a qualified clinical decision maker. When evaluating a patient's status, NEWS2 should be used in conjunction with clinical judgment [20]. Some patients who might develop increased oxygen requirements during their course in the hospital, may not have any additional significant increase in the NEWS2 score. However, this must trigger an escalation call to a competent clinician [20].

With the data of 814 patients, our study found mortality to be 9.09% in our tertiary care hospital. Mortality was greater within higher age groups and with associated comorbidities like hypertension (HTN) and diabetes mellitus (DM). NEWS2 score was found to be statistically significant in determining the outcome (death or discharge) in our study, with AUROC being 0.99. "Accuracy" is used when the class distribution is similar, while "F1-score" is a better metric when there are imbalanced classes, as in our case (74 deaths and 740 discharges). In most real-life classification problems, imbalanced class distribution exists and thus F1-score is a better metric to evaluate our model on. To minimize the incorrect prediction of mortality, we chose NEWS2 ≥ 6 as the ideal cut-off point for its slightly lesser but still significant sensitivity of 93.24% as compared to NEWS2 ≥ 5, and for having greater specificity of 98.91%, precision of 0.89, and F-1 score 0.91. A study on 66 patients by Myrstad et al., found that NEWS2 score ≥ 6 predicted severe disease (ICU admission) with 84.3% specificity and 80.0% sensitivity [25]. However, they concluded that larger prospective studies are required to confirm the cut-off point [25]. Another study with 110 patients done in South Korea by Jang et al. concluded that patients with NEWS2 ≥ 7 had a shorter survival time, however, the results cannot be generalized as it was a retrospective study conducted on a small population [17]. Our prospective study resolves these shortcomings as it was conducted on a larger sample size, thus reducing the chances of error and allowing more accurate prediction. In the pre-COVID period, a score of 5 to 6 indicated a medium risk of deterioration, while a score of 7 or more indicated a high risk of deterioration as per Royal College of Physicians standards [20]. However, in our study on COVID-19 patients, we found that a NEWS2 cut-off score ≥ 6 on admission had higher risk of mortality.

## Conclusions

Clinical judgment and careful monitoring are recommended throughout the course of treatment. NEWS2 score calculated in the emergency department helps us triage patients according to the care they should receive. Prompt management and timely monitoring should be warranted for patients with higher NEWS2 scores. In our study on COVID-19 patients, we found that a NEWS2 cut-off score ≥ 6 on admission had higher risk of mortality. Therefore, such patients should be continuously monitored by admitting them in intensive care units and emergency assessment by a team with critical care competencies should be made available at the earliest. Our study conducted on 814 patients identified NEWS2 to be an efficient tool for the early recognition of high-risk COVID-19 patients in the emergency department. The requirement of minimal equipment and ease of calculation will also allow remote hospitals to assign these patients to the most suitable care level, i.e., home quarantine, routine hospitalization, or intensive care.
